# Supplementary material for: Direct Derivatization in Dried Blood Spots for Oxidized and Reduced Glutathione Quantification in Newborns
Source: Antioxidants (Basel). 2022 Jun 14;11(6):1165. doi: 10.3390/antiox11061165 (PMC9219658; doi:10.3390/antiox11061165)
Supplement: Supplementary file 1 [file antioxidants-11-01165-s001.zip › antioxidants-1744181-supplementary.pdf]

Supporting Information

Direct derivatization in Dried Blood Spots for Oxidized and Reduced Glutathione Quantification in Newborns

Isabel Ten-Doménech [1], Álvaro Solaz-García [1], Inmaculada Lara-Cantón [1], Alejandro Pinilla-Gonzalez [1], Anna Parra-Llorca [1], Máximo Vento [1,2], Guillermo Quintás [3,4,*], and Julia Kuligowski [1,*]

[1] Neonatal Research Group, Health Research Institute La Fe, 46026 Valencia, Spain;
isabel_ten@iislafe.es (I.T.-D.); alvarosogar@gmail.com (Á.S.-G.);
inmaculada_lara@iislafe.es (I.L.-C.); alejandro_pinilla@iislafe.es (A.P.-G.);
parra_ann@gva.es (A.P.-L.); maximo.vento@uv.es (M.V.);
julia.kuligowski@uv.es
(J.K.)

[2] Division of Neonatology, University and Polytechnic Hospital La Fe, 46026 Valencia, Spain

[3] Health and Biomedicine, LEITAT Technological Center, 08225 Terrassa, Spain; gquintas@leitat.org (G.Q.)

[4] Unidad Analítica, Health Research Institute Hospital La Fe, 46026 Valencia, Spain

* Correspondence: gquintas@leitat.org (G.Q.); julia.kuligowski@uv.es (J.K.); Tel.: +34-961-246-661 (J.K.)

Materials and Methods

Preparation of stock, working and standard solutions

Individual stock solutions of glutathione (GSH) and GSH-(*glycine*-$^{13}C_2$,^{15}N) at a concentration of 10 mmol/L and 1 mmol/L, respectively, were prepared in phosphate buffer solution (PBS) (10 mmol/L, pH 7) containing *N*-ethylmaleimide (NEM) at a concentration of 100 mmol/L and kept at -20 °C. Individual stock solutions of oxidized glutathione (GSSG) and GSSG-D_{10} at a concentration of 5 mmol/L and 0.8 mmol/L, respectively, were prepared in H_2O (0.1% formic acid (FA), v/v) and kept at -20 ºC. By mixing appropriate volumes of these individual solutions, a working solution of 2 mmol/L GSH-NEM and 100 µmol/L GSSG in H_2O (0.1% FA, v/v) and an internal standard (IS) mixture of GSH-$^{13}C_2$,^{15}N-NEM and GSSG-D_{10} at 100 µmol/L each were prepared. Calibration curves of GSH-NEM and GSSG were prepared freshly on each measurement day by serial dilution in perchloric acid (PCA) (1%, v/v) with FA (0.1%, v/v) at concentrations ranging from 185 to 190000 nmol/L and from 9 to 9400 nmol/L, respectively.

Sample treatment according to previous developed method [20]

50 µL of blood were added to 10 µL of NEM 100 mmol/L in PBS and after 5 min of incubation at room temperature, 60 µL of cold PCA (8%, v/v) were added. Then, samples were thoroughly mixed and centrifuged at 10000 *g* for 15 min at 4 °C. Supernatant (10 µL) was added to 90 µL of H_2O (0.1% FA, v/v). Finally, 5 µL of IS solution containing isotopically labelled GSH-NEM and GSSG at 100 µmol/L were added.

Table S1. Acquisition parameters and main figures of merit of the LC-MS/MS method.

Analyte	m/z parent ion	Cone (V)	m/z daughter ion				Time window (min)	RT ± SD (min)	Calibration range (nmol/L)	Calibration curve fitting	R^2	LOD (nmol/L)	LOQ (nmol/L)
			Quant.	CE (eV)	Conf.	CE (eV)							
GSH-NEM	433.2	30	304.3	15	201.2	15	4.0 - 5.0	4.493 ± 0.005	185 - 190000	Quadratic	0.9996	14	185
GSSG	613.2	30	355.3	25	231.10	25	1.0 - 2.0	1.426 ± 0.007	9 - 9400	Linear	0.997	3	9
GSH-$^{13}C_2$,^{15}N-NEM	436.10	30	201.25	20	-	-	4.0 - 5.0	4.486 ± 0.005	-	-	-	-	-
GSSG-D_{10}	623.0	35	355.1	20	-	-	1.0 - 2.0	1.416 ± 0.006	-	-	-	-	-

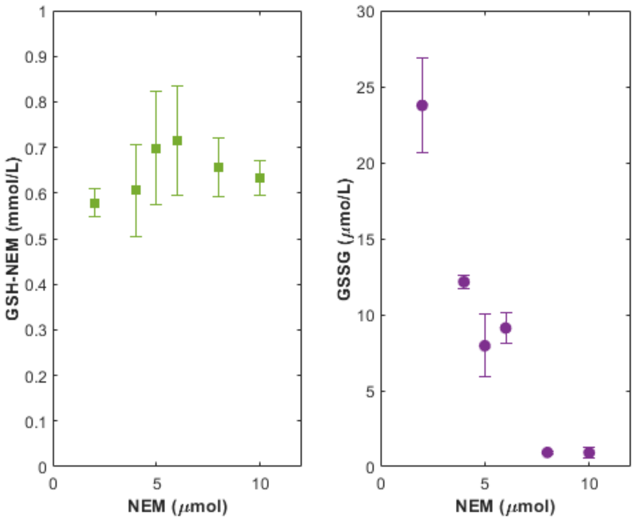

Figure S1. Determined GSH-NEM and GSSG concentrations in the same whole blood sample deposited on DBS paper cards pre-soaked with increasing amounts of NEM. Error bars represent standard deviation of replicates (N = 3).

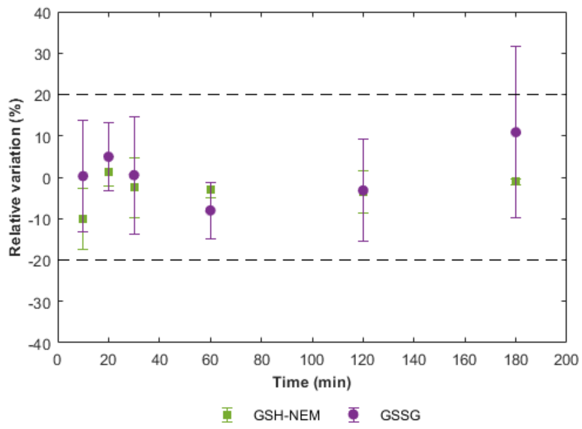

Figure S2. Relative variation in GSH-NEM and GSSG concentrations over time with respect to GSH-NEM and GSSG concentrations at t = 0 for the same DBS sample. Error bars represent standard deviation of replicates (N = 3).

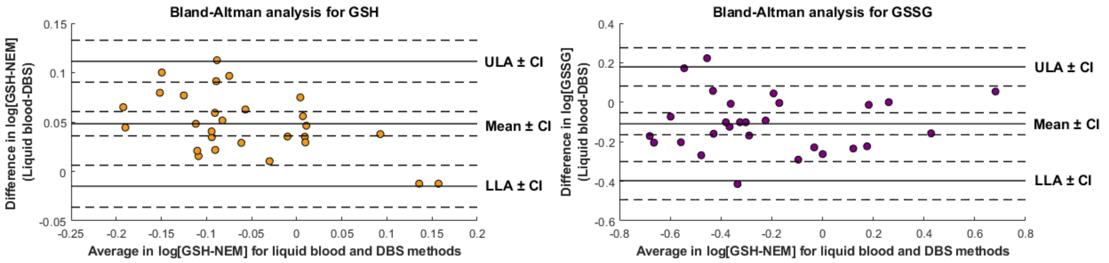

Figure S3. Bland-Altman plots with log-transformed data for the comparison between DBS method and liquid blood method for GSH-NEM and GSSG (N = 28). Note: CI, 95% Confident Interval; LLA, lower limit of agreement; ULA, upper limit of agreement.

Table S2. Characteristics of the study population.

Demographics	Term infants (N = 161)
Gender, N (%)	
Male	85 (53)
Female	76 (47)
GA median (5-95% CI)	39 (39-40)
Maternal age (5-95% CI)	33 (32-34)
Birth weight (g), mean (SD)	3200 (500)
Birth length (cm), mean (SD)	50 (2)
Birth HC (cm), mean (SD)	34.2 (1.3)
Mode of birth, N (%)	
Vaginal	69 (43)
C-section	92 (57)
1-min Apgar score, median (IQR)	9 (8-10)
5-min Apgar score, median (IQR)	10 (10-10)
Hematocrit[a], mean (SD)	49 (5)

[a] calculated from 61 out of 73 cord blood samples

Note: GA, gestational age; CI, confidence interval; SD, standard deviation; HC, head circumference; IQR, interquartile range.

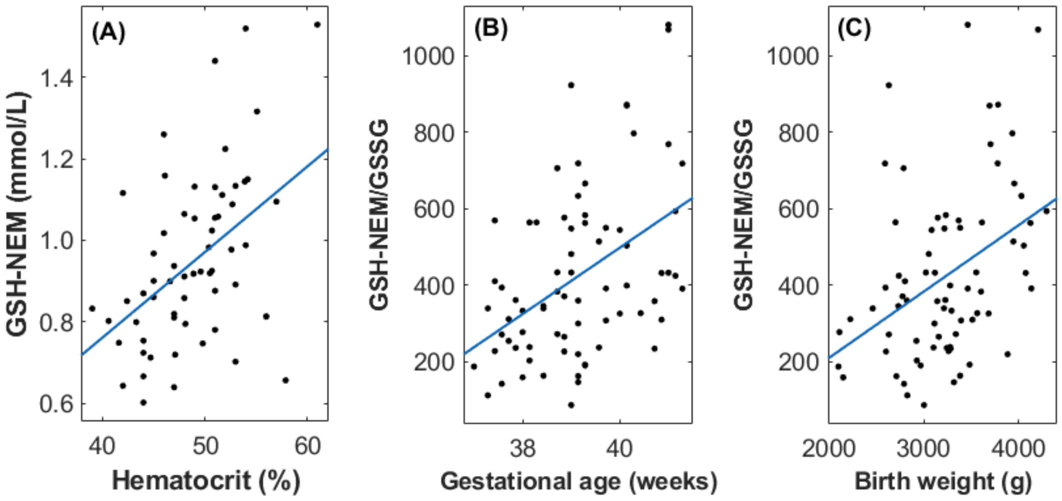

Figure S4. Linear fitting of GSH-NEM concentration in cord blood with hematocrit (r = 0.5; p-value < 0.001) (A); and GSH-NEM/GSSG with gestational age (r = 0.5; p-value < 0.001) (B), and birth weight (r = 0.4, p-value < 0.001) (C).

Table S3. GSH-NEM, GSSG, and GSH-NEM/GSSG median values (interquartile range) of cord blood and at discharge. Note: DBS samples were stored at 4 ºC for 24h (short term storage, STS) and at -20 ºC for 1 to 30 days (long term storage, LTS).

	Cord blood (N = 47)			At discharge (N = 51)		
Analyte	STS	LTS	*p*-value[a]	STS	LTS	*p*-value[a]
GSH-NEM (mmol/L)	0.9 (0.2)	1.0 (0.3)	0.0010	1.3 (0.4)	1.3 (0.4)	0.2
GSSG (µmol/L)	2 (2)	3 (2)	0.0003	4 (2)[b]	4 (2)[b]	0.015
GSH/GSSG	400 (300)	300 (200)	0.007	370 (150)[b]	330 (130)[b]	0.03

[a] Wilcoxon signed rank test for paired samples

[b] N = 48
